# TIS7 induces transcriptional cascade of methylosome components required for muscle differentiation

**DOI:** 10.1186/s12915-016-0318-6

**Published:** 2016-10-25

**Authors:** Andrea Lammirato, Katherin Patsch, Fabien Feiereisen, Karl Maly, Charity Nofziger, Markus Paulmichl, Hubert Hackl, Zlatko Trajanoski, Taras Valovka, Lukas A. Huber, Ilja Vietor

**Affiliations:** 1 Division of Cell Biology, Biocenter, Medical University Innsbruck, Innrain 80-82, A-6020 Innsbruck, Austria; 2 Division of Medical Biochemistry, Biocenter, Medical University of Innsbruck, Innrain 80-82, A-6020 Innsbruck, Austria; 3 Division of Bioinformatics, Biocenter, Medical University of Innsbruck, Innrain 80-82, A-6020 Innsbruck, Austria; 4 Institute of Pharmacology and Toxicology, Paracelsus Medical University, Strubergasse 21, A-5020 Salzburg, Austria

**Keywords:** TIS7, DNA-binding, Methyl transferase, Epigenetic regulation, ICln, MyoD, Myogenesis

## Abstract

**Background:**

TPA Induced Sequence 7 acts as a transcriptional co-regulator controlling the expression of genes involved in differentiation of various cell types, including skeletal myoblasts. We and others have shown that TIS7 regulates adult myogenesis through MyoD, one of the essential myogenic regulatory factors.

**Results:**

Here, we present data identifying ICln as the specific, novel protein downstream of TIS7 controlling myogenesis. We show that TIS7/ICln epigenetically regulate *myoD* expression controlling protein methyl transferase activity. In particular, ICln regulates MyoD expression via its interaction with PRMT5 by an epigenetic modification that utilizes symmetrical di-methylation of histone H3 on arginine 8. We provide multiple evidences that TIS7 directly binds DNA, which is a functional feature necessary for its role in transcriptional regulation.

**Conclusion:**

We present here a molecular insight into TIS7-specific control of MyoD gene expression and thereby skeletal muscle differentiation.

**Electronic supplementary material:**

The online version of this article (doi:10.1186/s12915-016-0318-6) contains supplementary material, which is available to authorized users.

## Background

Mouse TIS7, its rat PC4 [1], and human Interferon-Related Developmental Regulator 1 [2, 3] homologues were originally proposed to act as immediate early genes [1, 4]. TIS7 was found to be upregulated upon c-Jun activation and translocated to the nucleus [5], where it functions as a transcriptional co-regulator, activating [2] or repressing gene expression via interaction with transcription factors and histone deacetylase [6] complexes [5, 7, 8]. TIS7 protein has been shown to be involved in the regulation of differentiation processes in multiple cell types, e.g., neurons [4, 9], enterocytes [10], and myocytes [11, 12]. We have previously shown that TIS7 knockout (KO) mice are viable and fertile but suffer from delayed muscle regeneration upon muscle crush damage, which becomes even more apparent with increasing age [12]. At the cellular level, primary muscle satellite cells from TIS7 KO mice displayed significant differentiation deficiency [12]. This regeneration phenotype can be explained by reduced differentiation potential caused by decreased mRNA and protein expression levels of key muscle regulatory factor MyoD [12] in TIS7 KO muscle satellite cells (MSCs). We, together with others, have previously shown that TPA Induced Sequence 7 [7] is a positive regulator of myogenesis and of MyoD levels in particular. Although there is experimental evidence for TIS7-dependent regulation of multiple proteins related to the control of MyoD levels and its activity, e.g., depletion of the inhibitory effect of HDAC4 [2] or downregulation of NF-κB [13], an exact TIS7-dependent pathway regulating MyoD levels and thereby myogenesis remained thus far unknown.

Though there is indication that transcriptional regulation of myogenic regulatory factors (MRFs) myoD, myf5, and myogenin depends on the enzymatic activity of several histone modifying enzymes, such as protein arginine *N*-methyltransferase 5 [14–16], the mechanism regulating the particular choice of PRMT5 substrate in adult myogenesis is yet to be identified. PRMT5 was found in the methylosome complex with the protein ICln which, depending on its interacting partners, is also involved in transcriptional regulation (for review see [17]). ICln was shown to be responsible for the PRMT5 substrate specificity [18]. However, whether ICln levels correlate with the PRMT5 activity and mainly how they are regulated in the process of myogenesis, remains to be studied.

Herein, we investigated, at the molecular level, how TIS7 may affect skeletal muscle regeneration and the regulatory processes of myoblast differentiation, in particular, the exact mechanism by which TIS7 regulates MyoD levels. First of all, we identified that the TIS7 protein binds directly to DNA, which substantially clarifies its role in transcriptional regulation. We confirmed by several independent techniques the physical interaction between the TIS7 protein and regulatory elements of the ICln gene and even quantitatively determined the relationship between TIS7 binding and ICln transcriptional regulation. Next, we clarified the link between PRMT5 and regulation of myoD expression by showing that ICln levels determine binding of PRMT5 and thereby its methyl transferase activity on histone H3 Arg 8 on particular gene locus, in this case myoD. Finally, through in vivo experiments, we documented that the ectopic expression of either TIS7, ICln, or MyoD in MSCs derived from TIS7 KO mice rescued their diminished differentiation potential.

On the basis of our current data, we propose that TIS7 participates in the mechanism of epigenetic regulation of myogenesis via a novel player ICln, in particular by its transcriptional regulation.

## Results

### TIS7 controls myoD levels through epigenetic regulation of gene expression

**Fig. 1:**
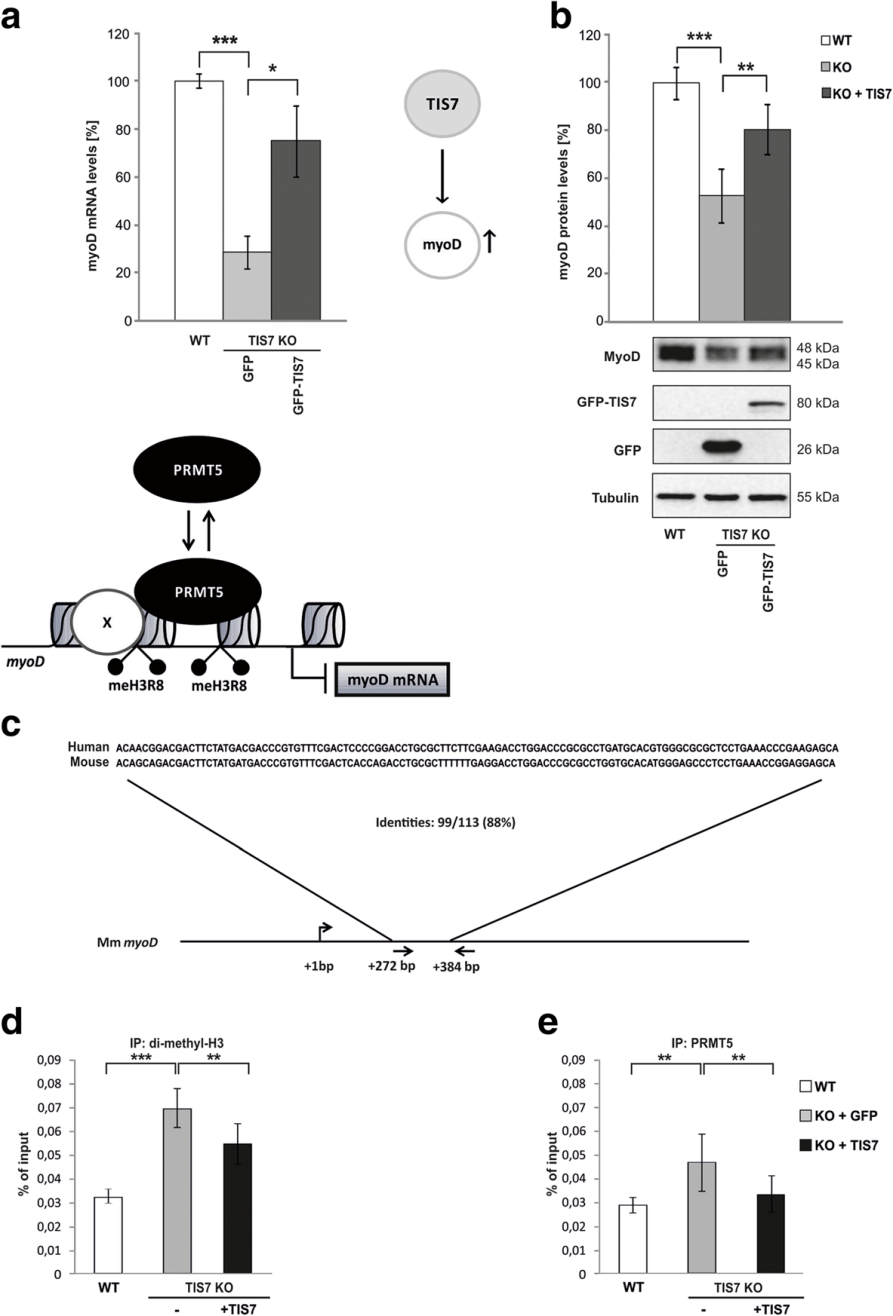
TIS7 epigenetically regulates MyoD expression. **a** TIS7 KO MSCs were transfected with indicated plasmids. Quantitative PCR analysis of *myoD* was normalized to GAPDH expression (n = 3 independent experiments). Values represent the mean ± SEM and resulting data were analyzed via two-tailed type 2 Student’s t-test, *** *P* < 0.001 and * *P* < 0.05. **b** Proliferating TIS7 KO MSCs were transfected with indicated plasmids and total cell lysates were probed with MyoD and control antibodies. Quantification (n = 3 biological replicates) was performed by normalization to the tubulin signal. Values represent the mean ± SD and resulting data were analyzed via two-tailed type 2 Student’s t-test, *** *P* < 0.001 and ** *P* < 0.01. Representative western blots are shown. ChIP analyses using chromatin from TIS7 WT or KO MSCs transfected with either GFP or GFP-TIS7 plasmid DNA. **c** Schematic outline of mouse *myoD* gene locus. Primer pairs used for ChIP assays are indicated by arrows. The alignment of human and mouse sequences amplified by the PCR is shown. Antisera directed against symmetrically di-methylated histone H3 at arginine 8 (**d**) or PRMT5 (**e**) were used for immunoprecipitation. Immunoprecipitated DNA was analyzed by qPCR in triplicates using primers specific for the mouse myoD regulatory region. ChIP with control rabbit IgG or 1.23 % of total chromatin (input) were used as controls. Signals were normalized to input chromatin and shown as percentage of input, ** *P* ≤ 0.001 and *** *P* < 0.00005

Myo5D and MEF2 families of transcription factors are key regulators of skeletal muscle differentiation [19, 20]. We and others have previously shown that MyoD levels in myoblasts depend on TIS7 expression [2, 12, 13]. Although the levels of several proteins related to myogenesis regulation (HDAC4, NF-kB) have been shown to be affected by the presence or absence of TIS7 [2, 13], the explicit mechanism by which TIS7 regulates MyoD expression is yet to be identified. Therefore, we decided to first analyze the ability of ectopic TIS7 expression to restore MyoD mRNA and protein levels in proliferating TIS7 KO MSCs. Consistent with previous data [12], the loss of TIS7 caused a significant downregulation of myoD mRNA levels, which was restored upon ectopic expression of TIS7 in KO MSCs (Fig. 1a). Western blot analyses of MyoD showed that (1) Myod D protein levels were significantly reduced in TIS7 KO MSCs (approx. 50 % of the WT value) and (2) significant rescue was achieved by TIS7 ectopic expression (approx. 80 %) (Fig. 1b).

### TIS7 regulates myoD expression by an epigenetic mechanism

Transcriptional co-regulator arginine methyltransferase PRMT5 was identified to be necessary for proper myogenesis in zebrafish embryonic development, where it specifically regulates myoD expression [21]. In adult mice, PRMT5 inactivation abrogated skeletal muscle regeneration [14], which resembled the TIS7 KO mouse phenotype reported by us [12]. Therefore, we decided to investigate whether the PRMT5-dependent epigenetic control mechanism of *myoD* expression is affected by TIS7 KO. We first tested the presence of arginine 8 dimethylated histone H3 on the *myoD* gene. For this purpose, we performed chromatin immunoprecipitation (ChIP) using specific anti-histone H3 (sym-dimethyl Arg8) antibodies followed by quantitative PCR detection of the *myoD* regulatory region (Fig. 1c). Our data showed that there was a significant increase in symmetrical dimethylation of histone H3 on *myoD* in TIS7 KO when compared to wildtype (WT) MSCs (*P* < 0.00005; Fig. 1d), although the total amounts of histone H3 were comparable (Additional file 1: Figure S1A). The increase in this modification, which is generally related to transcriptional inhibition, explained reduced *myoD* expression in TIS7 KO MSCs. Importantly, the ectopic expression of TIS7 in KO MSCs significantly (*P* ≤ 0.001) reduced symmetrical dimethylation of histone H3 on *myoD* to levels comparable with the WT MSCs. Next, we analyzed by ChIP whether this difference was directly dependent on PRMT5 recruitment to the *myoD* regulatory region. As expected, PRMT5 occupancy on the *myoD* gene was significantly higher in TIS7 KO when compared to WT MSCs. As in the case of H3 methylation, we found that the ectopic expression of TIS7 significantly reduced PRMT5 binding to *myoD* gene in KO MSCs (*P* ≤ 0.001; Fig. 1e).

### ICln expression is downregulated in TIS7 KO MSCs

**Fig. 2:**
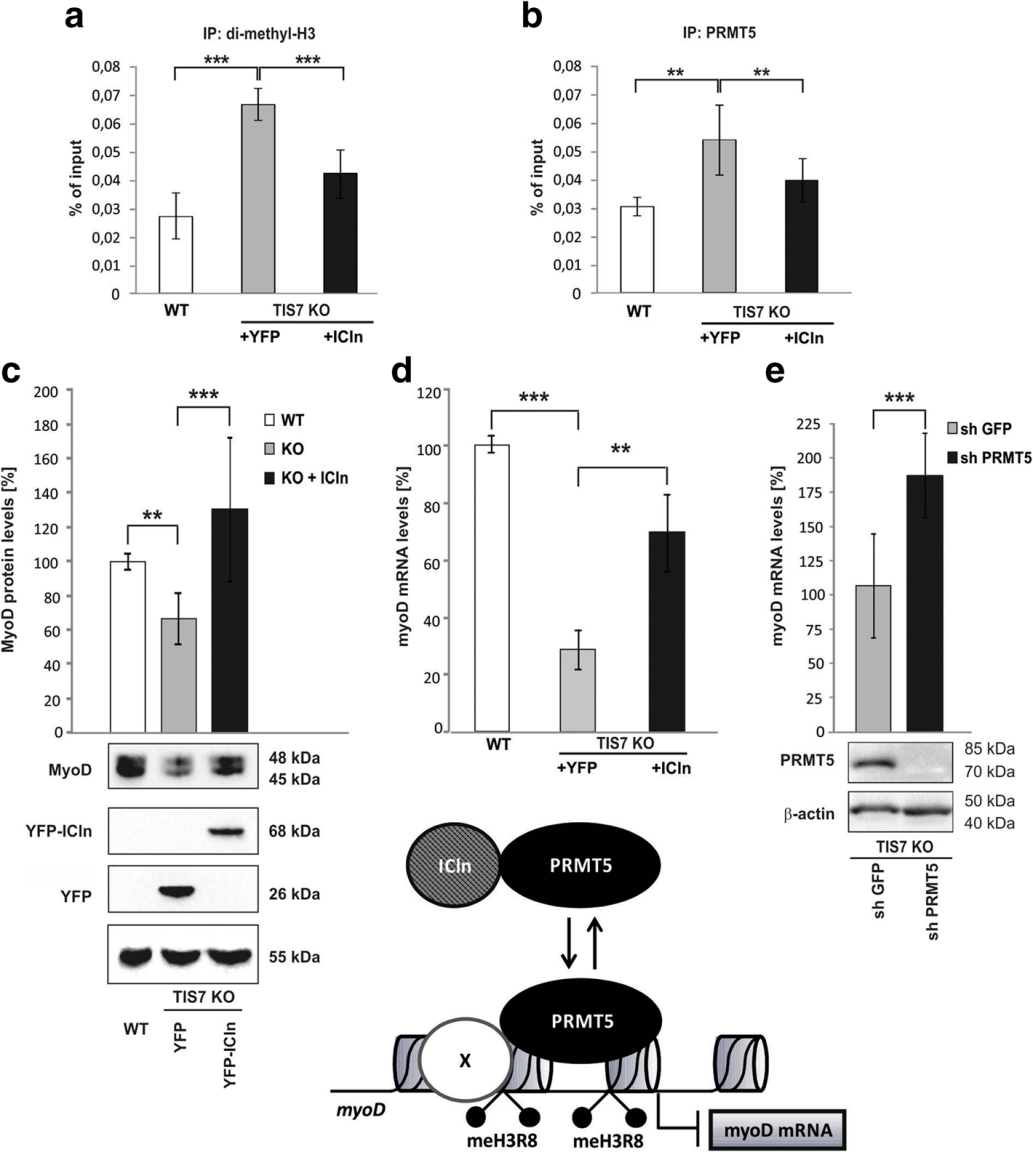
ICln affects in vivo methylation of histone H3 and binding of PRMT5 to myoD locus, resulting in increased *myoD* RNA and protein levels. ChIP analysis using chromatin from TIS7 WT or KO MSCs transfected with either YFP or YFP-ICln plasmids. Antisera directed against symmetrically dimethylated histone H3 at arginine 8 (**a**) or PRMT5 (**b**) were used for immunoprecipitation. Immunoprecipitated DNA was analyzed in triplicate by qPCR using primers to amplify the mouse myoD regulatory region. ChIP with control rabbit IgG or 1.23 % of total chromatin (input) were used as controls. Signals were normalized to input chromatin and shown as percentage of input. ** *P* ≤ 0.001 and *** *P* < 0.00005. **c** TIS7 KO MSCs were transfected with indicated plasmids and total cell lysates were probed with anti-MyoD and anti-YFP antibodies. Quantification (n = 3 biological replicates) was performed by normalization to the tubulin signal. Representative immunoblots are shown. **d** TIS7 KO MSCs were transfected with the indicated plasmids. Quantitative PCR analysis of *myoD* was normalized to *GAPDH* expression (n = 3 biological replicates and ** *P* < 0.01). **e** PRMT5 knockdown significantly increased *myoD* RNA levels in TIS7 KO MSCs transduced with sh PRMT5-expressing lentivirus. *myoD* RNA levels were measured by qPCR and PRMT5 protein levels were documented by immunoblots with indicated antibodies

To understand how TIS7 may affect PRMT5-specific function in myogenesis we analyzed the effect of ectopic expression of a known regulator of PRMT5, namely ICln (37 kDa) [18, 22, 23] in TIS7 WT and KO MSCs. ICln protein forms a complex with PRMT5 [24] that regulates its substrate specificity [25]. Therefore, we analyzed whether PRMT5 epigenetic regulation of *myoD* transcription was related to ICln levels. Under an identical experimental setup as described above (Fig. 1d, e), we analyzed chromatin isolated from TIS7 WT and KO MSCs, transiently transfected either with control YFP or with a YFP-ICln construct. Immunoprecipitation with both anti-histone H3 (sym-dimethyl Arg8) and anti-PRMT5 antibodies revealed an increase in Arg8 dimethylated histone H3 and PRMT5 bound to myoD regulatory elements in TIS7 KO MSCs, respectively (Fig. 2a, b). Constitutive expression of ICln significantly (*P* ≤ 0.0001) decreased the amount of symmetrically dimethylated H3 Arg8 (*P* ≤ 0.001) on *myoD* regulatory elements; this effect could be explained either by increased PRMT5 enzymatic activity or changes in its binding to chromatin. Two different independent methylation assays unveiled that the presence of ICln had no effect on total PRMT5 enzymatic activity both in vitro and in vivo (Additional file 1: Figure S1C and D). We also could not detect any difference in subcellular distribution of PRMT5 (data not shown). However, our ChIP experiments revealed that the ectopic expression of ICln substantially decreased the symmetrically dimethylated arginine 8 histone H3 and PRMT5 recruitment to myoD regulatory elements (Fig. 2a, b). The level of chromatin-associated PRMT5 in ICln-complemented TIS7 KO MSCs was similar to that observed in WT cells. Next, we checked whether ICln ectopic expression in TIS7 KO MSCs rescued the myoD expression. As shown in Fig. 2c, the ectopic ICln expression rescued MyoD protein levels due to increased *myoD* transcription, as documented by elevated *myoD* mRNA (Fig. 2d). Thereafter, we asked ourselves whether PRMT5-mediated methylation of the myoD regulatory region is solely responsible for TIS7/ICln-mediated regulation of myoD transcription. Therefore, we generated, by lentiviral transduction and antibiotic selection, a stable and specific PRMT5 knockdown in TIS7 KO MSCs (Fig. 2e and Additional file 1: Figure S1B). qPCR analysis of myoD levels in proliferating TIS7 KO MSCs did not show any difference between control sh GFP and sh PRMT5 expressing cells (data not shown). However, after 7 days in differentiation medium, at a time point when WT TIS7 MSCs fully differentiate, we identified an 80 % increase in myoD RNA levels of sh PRMT5-expressing TIS7 KO MSCs (Fig. 2e). Based on these results we concluded that PRMT5-dependent methylation of histone H3 bound to myoD regulatory elements is not the main regulatory mechanism in proliferating TIS7 deficient cells, suggesting that other TIS7-dependent mechanisms may contribute to this regulation. However, PRMT5-regulated methylation significantly affected myoD expression during the differentiation of MSCs.

We thus conclude that ICln regulates MyoD expression in MSCs by changing the epigenetic status of chromatin through functional interference with PRMT5.

**Fig. 3:**
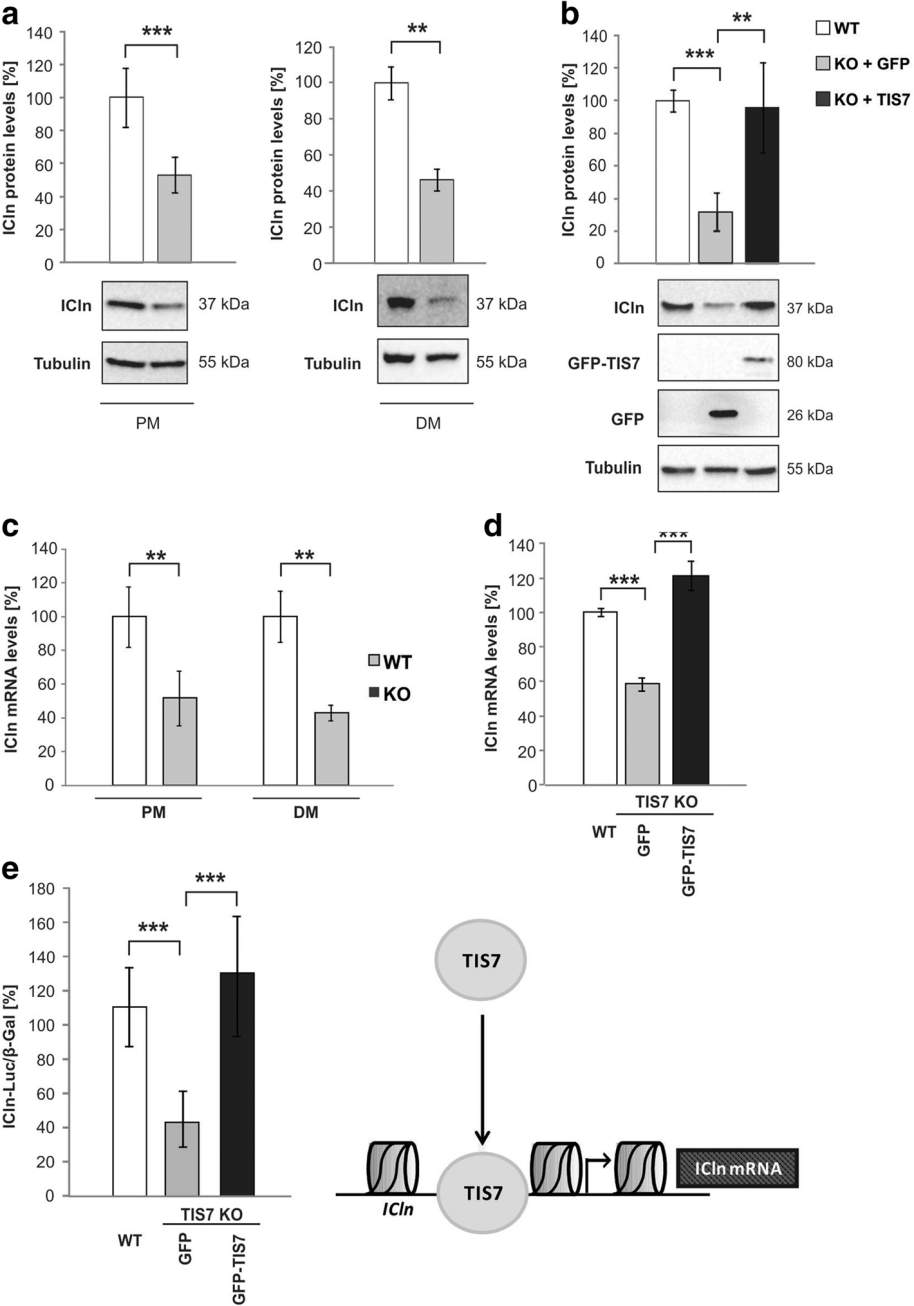
TIS7 regulates ICln protein and RNA levels. **a** Quantitative analyses of ICln protein expression in proliferating (PM; n = 4 biological replicates) and differentiating (DM; n = 3 independent experiments) MSCs. Equal amounts of total cell lysates were probed by immunoblotting with anti-ICln antibodies and normalized by tubulin. **b** TIS7 ectopic expression rescued ICln protein levels in TIS7 KO MSCs. Proliferating TIS7 KO MSCs were transfected with GFP plasmid as a control or with GFP-TIS7 plasmid constructs and total cell lysates were probed with the indicated antibodies. Quantification (n = 3 biological replicates) was based on tubulin signal. Representative western blots are shown. **c** Quantitative PCR analysis of *ICln* was normalized to *GAPDH* expression (n = 3 biological replicates). **d** TIS7 overexpression rescued *ICln* RNA levels in TIS7 KO MSCs. Proliferating TIS7 KO MSCs were transfected with indicated plasmids and ICln quantitative PCR was performed as in panel c. **e** TIS7 transcriptionally regulates ICln levels. Proliferating WT MSCs were transiently co-transfected with full length ICln promoter-luciferase reporter construct, β-galactosidase expression plasmid, and GFP plasmid as a control or GFP-TIS7 plasmid constructs. Luciferase values were normalized to β-galactosidase values. In all graphs, values represent the mean ± SD and resulting data were analyzed via two-tailed type 2 Student’s t-test, *** *P* < 0.001, ** *P* < 0.01, and * *P* < 0.05

The next question raised was whether there were significant differences in ICln levels between TIS7 KO and WT MSCs. Western blot analyses of several independent biological experiments (n ≥ 3) revealed that ICln protein levels were significantly reduced in both proliferating (PM) and differentiating (DM) KO MSCs (to 53 % and 46 %, respectively) (Fig. 3a) when compared to TIS7 WT MSCs. Ectopic expression of TIS7 restored the ICln protein levels up to 90 % of the WT values, suggesting the role of TIS7 in its regulation (Fig. 3b).

**Fig. 4:**
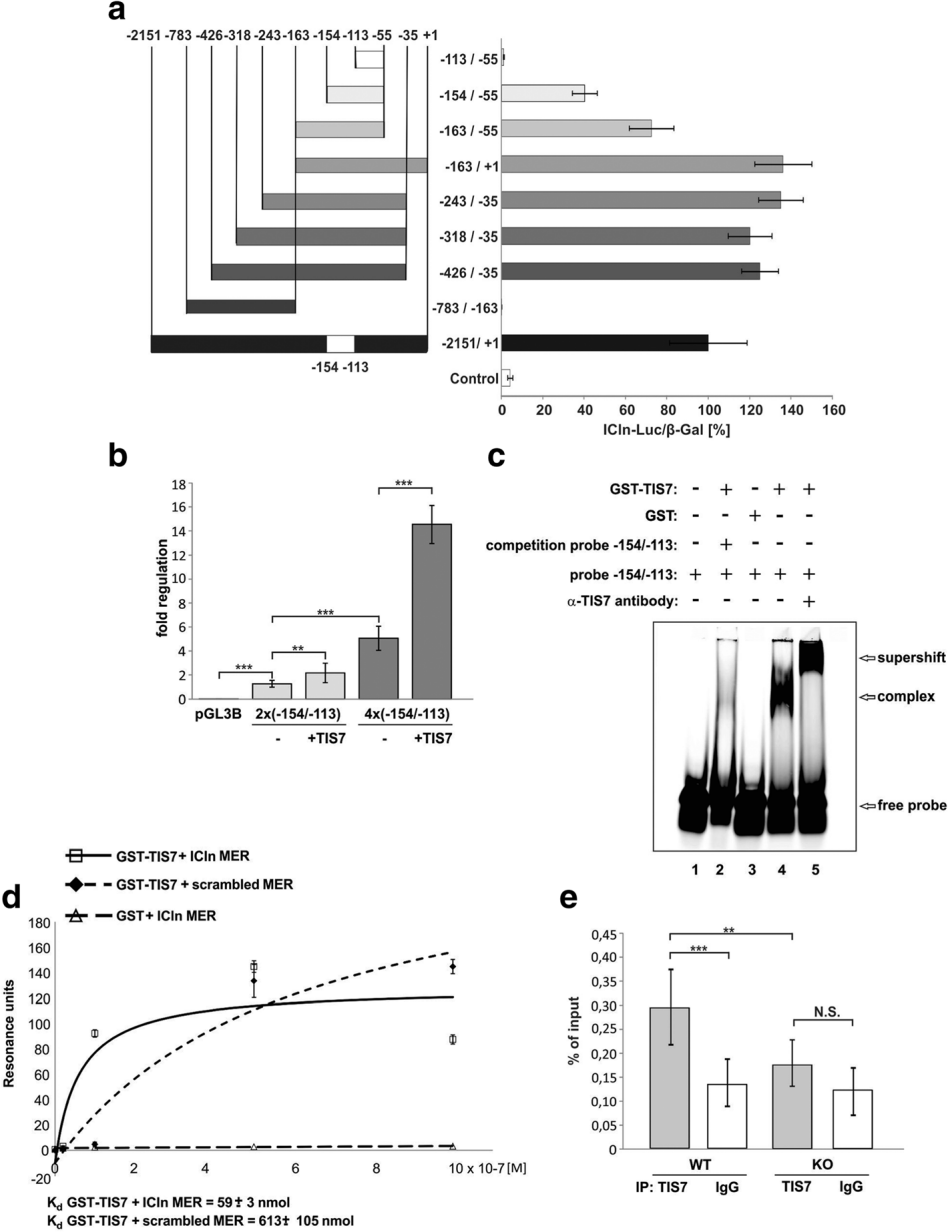
A defined 42 nucleotide stretch within the ICln promoter represents the minimal region essential for TIS7-regulated ICln transcription. **a** Proliferating TIS7 WT MSCs were transiently co-transfected with indicated ICln promoter-luciferase reporter constructs and with β-galactosidase expression plasmid for transfection control. The activity of the WT full length ICln promoter-driven luciferase reporter was set as 100 %. Values represent the mean ± SEM of at least three biological replicates. **b** Activities of luciferase reporter constructs driven by two or four (–154/–113) repeats from ICln promoter. Bars marked + TIS7 represent samples co-transfected with GFP-TIS7 plasmid vector. Control samples were co-transfected with GFP plasmid. **c** TIS7 binds to the minimal essential region necessary for the TIS7-regulated transcription of ICln. GST (lane 3) or GST-TIS7 (lanes 2, 4, and 5) recombinant proteins were incubated with the fluorescently labeled oligonucleotide probe containing the –154/–113 sequence of the ICln promoter. **d** Specific TIS7 binding to ICln minimal essential region (MER) determined by surface plasmon resonance measurements (SPR). Oligonucleotides representing either the ICln MER or its scrambled version were incubated with increasing concentrations of GST-TIS7 or GST alone as indicated. SPR steady-state response values (single values) are presented as a function of protein concentration (lines). K_d_ = dissociation constant. **e** ChIP analysis of TIS7 binding to the ICln promoter region. Chromatin from TIS7 WT and KO MSCs was immunoprecipitated as above using either specific antibody against TIS7 or control rabbit IgG

We observed the same trend also in mRNA levels where ICln expression was approximately 50 % lower in both PM and DM TIS7 KO MSCs (Fig. 3c). Similarly to protein, the level of ICln mRNA was significantly upregulated by TIS7 ectopic expression (Fig. 3d), indicating possible TIS7 involvement in ICln transcriptional regulation. To test this, we transiently transfected WT and TIS7 KO MSCs with an ICln promoter-driven reporter construct and measured the luciferase activities. These were reduced in TIS7 KO MSCs and efficiently rescued by ectopic expression of TIS7 (Fig. 3e) showing that the activity of ICln promoter is dependent on TIS7. Since little is known about *ICln* gene regulation [26], we applied luciferase assays using reporter constructs possessing various promoter truncations. These experiments revealed a 41 nt sequence (–154 to –113) representing a minimal essential region required for ICln transcriptional activity in WT MSCs (Fig. 4a).

### TIS7 directly binds to and activates ICln promoter

Next, we tested whether this 41 nt sequence was sufficient to regulate ICln expression in a TIS7-dependent manner. We generated a luciferase reporter construct, which contained two repeats of the 41 nt sequence (–154 to –113) of the ICln promoter upstream of the luciferase gene. WT MSCs transfected with this construct displayed increased luciferase activity when compared to the empty pGL3B luciferase vector (*P* > 0.001). This basal activity was significantly induced (*P* > 0.001) by co-transfection with TIS7 (Fig. 4b). Even stronger luciferase activity was found when four repeats of the 41 nt sequence were cloned upstream of the luciferase gene (5.1-fold induction reporter only and 14.5-fold induction when co-transfected with TIS7). These data demonstrated the functional relevance of this minimal essential region for TIS7-mediated activation of ICln transcription. Using bioinformatics prediction software (DISIS (www.predictprotein.org), BinDN+ (http://bioinfo.ggc.org/bindn+/), DP-Bind (http://lcg.rit.albany.edu/dp-bind/), and DNABindR (http://turing.cs.iastate.edu/PredDNA/); Additional file 2: Figure S2) we identified, with 70–80 % probability, three distinct DNA-binding domains in the TIS7 protein. This prediction suggested that the TIS7 protein may directly bind DNA. We tested this possibility by an electrophoretic mobility shift assay using an oligonucleotide probe containing the (–154 to –113) sequence of the ICln promoter. As shown in Fig. 4c, we detected a specific band representing a complex of GST-TIS7 with a bound fluorescently labeled oligonucleotide probe (lane 4). The specificity of this complex was confirmed by the fact that this complex was not formed when the probe was incubated with GST alone (lane 3). Additionally, TIS7 binding to the labeled probe was specifically blocked by pre-incubation with a 20-fold access of a non-labeled “cold” oligonucleotide (lane 2). The presence of TIS7 in this DNA complex was further confirmed by pre-incubation with a TIS7-specific antibody, which caused a “supershift”, i.e., further slowed the mobility of the complex on the native polyacrylamide gel (lane 5). In addition, we have quantified the specific binding of TIS7 to this DNA oligonucleotide using surface plasmon resonance. The measurements showed that (1) the protein binding moiety is specific (recombinant GST protein did not bind to the oligonucleotide probe) and (2) the TIS7-binding was sequence specific because there was a 10-fold difference in Kd values between the native (–154 to –113) ICln promoter (Fig. 4d) and “scrambled” sequences. Using ChIP analysis, we investigated whether TIS7 binds a native ICln promoter in MSCs. We found that TIS7 was bound to the ICln promoter in TIS7 WT but not KO MSCs (Fig. 4e). The specificity of TIS7 binding was confirmed using control rabbit IgG.

Based on the results of these analyses we conclude that TIS7 directly binds ICln promoter DNA and thereby positively regulates ICln transcription in MSC cells.

### TIS7 regulates myotube formation through ICln and MyoD expression

**Fig. 5:**
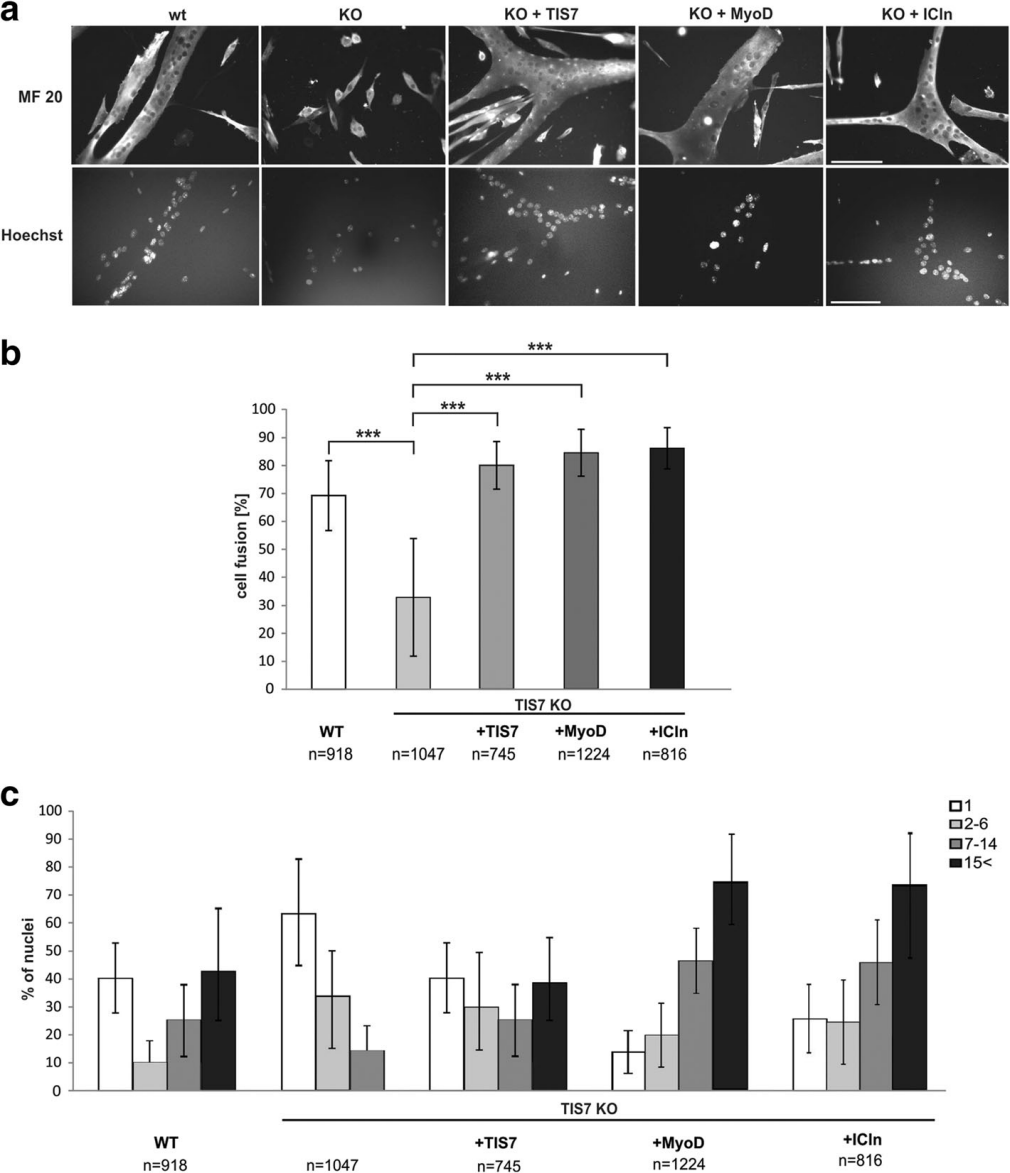
MSC differentiation is regulated by MyoD, ICln, and TIS7. **a** TIS7 WT and KO MSCs were plated at the same cell density. Proliferating TIS7 KO MSCs were transfected with indicated plasmids and 6 h later the differentiation was induced. After 5 d of differentiation, cells were fixed, stained with anti-myosin heavy chain antibody MF 20, and the nuclei were visualized using Hoechst. Scale bar = 100 μm. **b** Percentage of MSC fusion was calculated as the number of nuclei localized in multinucleated (n > 2) myotubes defined by MF20 staining compared to the total number of nuclei. Vertical bars denote the mean ± SD and were calculated from independent biological replicates (n = 3). The indicated number of nuclei was evaluated. Resulting data were analyzed via two-tailed type 2 Student’s t-test, *** *P* < 0.001. **c** Quantitative analysis of cells with the indicated nuclei numbers per cell defined by myosin staining. This analysis showed a significant decrease in the number of multinucleated cells and a completely missing category of cells with more than 15 nuclei in TIS7 KO MSCs when compared to TIS7 WT MSCs. Ectopic expression of TIS7, MyoD, or ICln significantly increased the number of multinucleated cells, mainly those with more than 15 nuclei in TIS7 KO MSCs when compared to TIS7 WT MSCs

To understand how TIS7 regulates skeletal muscle differentiation, we characterized in detail the morphological differences between primary WT and TIS7 KO MSCs. In general, proliferating MSCs could be induced to differentiate into mature myotubes within 5–7 days. Although plated at the same density, while 70 % of WT MSCs differentiated into myotubes, only 30 % of TIS7 KO MSCs formed myotubes (Fig. 5a, b). This defect was completely restored by ectopic TIS7 expression. Importantly, the ectopic expression of MyoD in TIS7 KO MSCs led to full recovery of the differentiation deficit and restored myotubes formation in these cells (Fig. 5a, b). Because of TIS7-dependent regulation of ICln we investigated whether ICln can act downstream of TIS7 to regulate myogenesis. Ectopic ICln expression in TIS7 KO cells rescued myotube formation in TIS7 KO MSCs, similar to the effects of TIS7 or MyoD overexpression (Fig. 5a, b).

A more detailed quantitative analysis revealed that the majority of TIS7 KO MSCs (>50 %) remained mononucleated and the very large myotubes containing more than 15 nuclei were completely missing in TIS7 KO MSCs (Fig. 5c). Ectopic TIS7 expression rescued the appearance of multinucleated cells, mainly the category of myotubes with more than 15 nuclei. Even more interestingly, the ectopic expression of MyoD reverted the distribution of different groups of multinucleated myotubes similar to that of TIS7 WT MSCs and recovered in TIS7 KO MSCs the missing group of very large myotubes with more than 15 nuclei (Fig. 5c). Similarly, the increased number of multinucleated cells and, specifically, the formation of very large myotubes with more than 15 nuclei (Fig. 5c) were observed in ICln-transfected cells. MyoD as a crucial myogenic regulatory factor is responsible for the myoblast differentiation leading to myotube formation and, based on data shown here, we concluded that TIS7 regulates ICln transcription and thereby MyoD levels responsible for reversion of the TIS7 KO phenotype in MSCs.

## Discussion

In this study, we show that ICln, a protein with multiple physiological functions that depend on its subcellular localization and mainly on its protein interacting partners [17], represents a novel regulator of skeletal myoblast differentiation. We identified that TIS7 transcriptionally regulated ICln via direct binding to its promoter and, secondly, that ectopic expression of ICln rescued the differentiation deficiency of TIS7 KO MSCs. ICln controlled muscle differentiation through the MyoD pathway since ICln upregulated MyoD levels. Our further results presented here provide evidence for the epigenetic regulation of *myoD* transcription by ICln.

Adult muscle regeneration depends on de novo myogenesis, which requires activation and commitment of quiescent satellite cells. Muscle regeneration ability is regulated by and depends on the differentiation potential of MSCs [27–29]. This is assured by the interplay of MRFs MyoD, myogenin, Myf5, and Myf6 (MRF4, herculin) [19, 30, 31]. MyoD and Myf5 are required for myogenic determination [32], whereas myogenin and Myf6 are markers of terminal differentiation [29]. Muscle damage induces MSC proliferation followed by differentiation resulting in the formation of new muscle fibers. MyoD is required in both processes; however, myoblast differentiation initiation and progression is governed by an interplay of MyoD with other muscle-specific factors, e.g., Myf5 and myogenin [33]. MyoD KO mice indicate a unique requirement of MyoD during adult muscle regeneration rather than during embryonic development [29]. We found that the myoD regulation at this stage of myogenesis is tightly regulated by the TIS7/ICln/PRMT5 axis. Depletion of TIS7 or reduced level of ICln caused the epigenetic inhibition of myoD leading to the general differentiation deficiency phenotype characterized by diminished myoblast fusion. These findings indicate that the maintenance of specific MyoD levels by the TIS7/ICln/PRMT5 axis is critical for the induction and early progression of myoblast differentiation. It remains to be clarified whether this pathway is responsible for the regulation of other MRF genes.

Myogenic gene expression is affected by chromatin remodeling enzymes such as histone acetylases and deacetylases, histone lysine methyltransferases, SWI/SNF family of ATP-dependent enzymes, and protein arginine methyltransferases [34, 35]. Among them, PRMT5 was identified in several independent studies as one of the critical epigenetic regulators of myogenesis. PRMT5 was shown to control the expression of the MRFs myogenin, MyoD, and Myf5 in zebra fish embryos [21]. It was also shown that PRMT5 is involved in transcriptional regulation of various other myogenic genes via symmetric arginine dimethylation of histones and components of the transcriptional machinery [15, 21]. PRMT5 facilitates the recruitment of Brg1-ATPase, associated with Swi/Snf chromatin remodeling enzymes, and MyoD to the promoter of myogenin via H3 Arg8 dimethylation resulting in upregulation of myogenin transcription [15]. The same H3 Arg8 modification by PRMT5 exerts transcriptional repression effect on the p21 gene that facilitates MSC proliferation and expansion, which has a positive impact on the process of muscle regeneration. As a part of the methylosome, PRMT5 also methylates and facilitates the recruitment of Sm proteins to the survival motor neuron complex leading to its activation [22, 36]. Interestingly, the loss of survival motor neuron-specific activity is observed in primary myoblasts from patients with the autosomal recessive disorder spinal muscular atrophy. These myoblasts display a strong fusion deficiency, implying a primary muscle defect along with the well-described neuronal degeneration [37, 38]. We present here new evidence for a contrasting, but not conflicting, function of PRMT5 in the regulation of myogenesis that is associated with inhibition of myoD expression. PRMT5 represses myoD expression using a similar epigenetic mechanism described for p21 gene that is mediated through H3Arg8 symmetrical dimethylation at the myoD locus. This inhibits induction and early progression of MSC differentiation into myotubes, which could represent the mechanism that together with p21 inhibition [14] contributes to the maintenance of the proliferative potential of adult MSCs. In this scenario, PRMT5 would allow efficient accumulation of a critical MSC mass that could be committed to differentiation upon receiving specific stimuli. The open question, however, remains how this PRMTR5 function is controlled in MSCs. It has been shown before that PRMT5-specific cellular functions are regulated by its interacting partners [39]. ICln, the component of the methylosome complex, was shown to directly interact with PRMT5 and change its substrate specificity. In the presence of ICln, PRMT5 methylation of Sm proteins is stimulated, but methylation of histones is inhibited [23]. Consistent with these studies, we also observed that ICln ectopic expression inhibited methylation of histone H3 in MSCs. This was achieved via inhibition of PRMT5 binding to the myoD gene (Fig. 2b) rather than changing PRMT5 enzymatic activity (Additional file 1: Figure S1C and D). The recruitment of PRMT5 to the regulatory elements of genes needs complex formation with DNA-binding or chromatin-associated adaptor proteins [40, 41]. We propose that formation of such a complex on the *myoD* gene is prevented by the interaction of PRMT5 with ICln. Similarly, formation of an alternative PRMT5 complex has been described for protein RioK1, which competes with ICln for binding to PRMT5 [18]. This would lead to diminished H3 Arg8 methylation thereby de-repressing myoD expression.

ICln has been found to stimulate PRMT5-mediated Sm protein methylation and at the same time to inhibit the nuclear function of PRMT5, namely methylation of histones [23]. Silencing of ICln was shown to cause α-motor axon degeneration [42], as seen in patients suffering from spinal muscular atrophy. There is a substantial body of knowledge on ICln functions, its interaction with other proteins and subcellular localization. However, the regulation of ICln gene expression remains to be solved. Here, we found a mechanism related to myogenesis where TIS7 transcriptionally activates ICln through binding to the newly identified regulatory element in the ICln gene. Thus, we propose that myoD expression in MSCs is epigenetically regulated via ICln/PRMT5 downstream of TIS7. This notion is supported by the following facts: (1) deletion of PRMT5 prevented MSC differentiation [14], which was a phenotype strikingly reminiscent of TIS7 KO MSCs; (2) PRMT5-specific symmetrical dimethylation of H3 Arg8 at the myoD gene was enhanced in TIS7 KO MSCs; and (3) TIS7 or ICln ectopic expression reduced H3 Arg8 methylation at the *myoD* gene and rescued the muscle differentiation deficiency in TIS7 KO MSCs. Furthermore, overexpression of PRMT5 alone in TIS7 KO MSCs did not rescue their fusion (data not shown), which was consistent with the inhibitory role of PRMT5 in MSC-specific myoD regulation.

## Conclusion

**Fig. 6:**
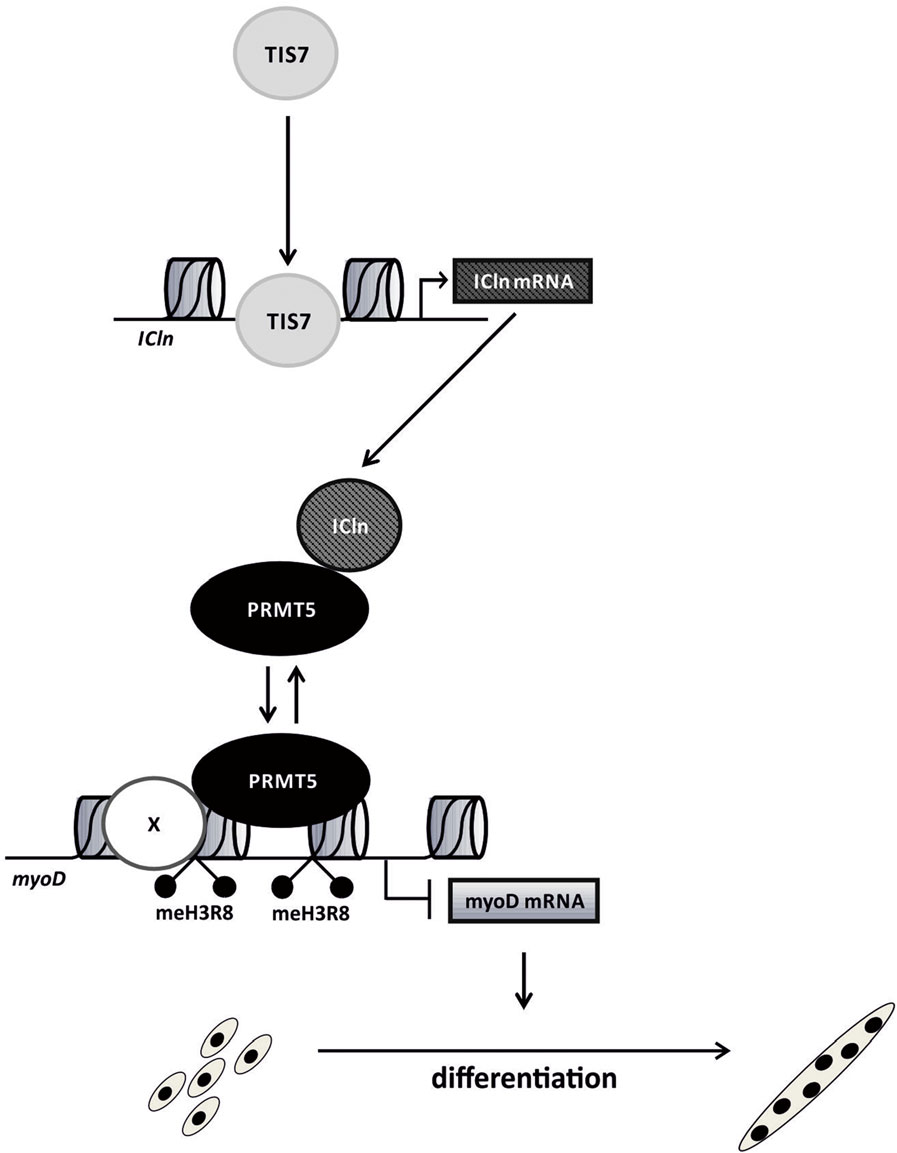
Model of myogenesis regulation by the TIS7/ICln/PRMT5 pathway. In skeletal muscles, TIS7 transcriptionally regulates ICln. The accumulated ICln interacts with PRMT5 and prevents its recruitment to chromatin-bound complexes on the myoD gene. This regulates PRMT5-mediated symmetrical dimethylation of histone H3 Arg8 and myoD expression. The regulated myoD level affects the balance between proliferation and differentiation of MSCs

As summarized in Fig. 6, we propose here a novel model for TIS7 function in myogenesis, where TIS7 upregulates ICln transcription in MSCs via direct binding to the regulatory element in the promoter of this gene. Increase in ICln transcription leads to accumulation of ICln protein in the cytoplasm and nucleus. The increased amount of ICln protein inhibits PRMT5 recruitment to the myoD locus and prevents the methylation of histone H3 at Arg 8. Decreased methylation de-represses myoD expression, which is critical for MSC differentiation. It is likely that PRMT5 controls MyoD levels to balance the proliferation and differentiation potential of MSCs. Thus, the components of this pathway and mainly their regulation could be seen as targets for development of new therapeutic approaches for treatment of skeletal muscle diseases.

## Methods

### Isolation and cultivation of primary muscle satellite cells (MSCs)

Primary MSCs were isolated from lower hind limb muscle of 2- to 3-month old TIS7 WT and KO mice as described previously [43]. The generation of the TIS7 KO mouse, cell culture conditions, and reagents were described before [12]. Briefly, cells were proliferated in Ham’s F-10 medium without glutamine, supplemented with 20 % non-heat-inactivated fetal calf serum (FCS gold, GE Healthcare) and penicillin-streptomycin (GE Healthcare); 2.5 ng/mL recombinant bFGF-basic (PeproTech) was added freshly. Cells were differentiated in high-glucose DMEM, supplemented with 2 % non-heat-inactivated horse serum and penicillin-streptomycin (GE Healthcare). MSCs were grown on cell culture dishes coated with collagen from calf skin (Sigma).

### GST-TIS7 purification and pull-down assays

GST-TIS7 was expressed in *E. coli* BL21 by addition of IPTG to 0.1 mM final concentration and incubation at 20 °C overnight. The recombinant protein was purified using a GSTrapFF column with glutathione Sepharose beads (GE Healthcare). Elution was initiated with lysis buffer (50 mM Tris-HCl pH 8, 150 mM NaCl, 10 μg/mL aprotinin, 1 μg/mL pepstatin, 1 μg/mL leupeptin, 0.4 mM Pefablock SC containing 10 mM glutathione (Sigma)). Additional purity was achieved via gel filtration, using a Sepharyl S200 column (GE Healthcare). The GST-TIS7 sample was dialyzed against 50 mM Tris pH 7.4, 150 mM NaCl.

### Immunofluorescence microscopy

Cells were fixed with 4 % paraformaldehyde, permeabilized with 0.2 % Triton X-100 PBS and blocked with 1 % bovine serum albumin, 5 % horse serum, and 15 % goat serum (Thermo Fisher Scientific) in PBS. Samples were incubated with primary antibodies for 1 h, with secondary antibodies for 30 min and mounted in Mowiol (Merck Millipore). Images were acquired using an AxioImager M1.2 microscope (Carl Zeiss) and processed with AxioVision Release 4.5 SP1 software.

### Preparation of MSC total cell lysates and immunoblotting

MSC lysates were prepared as previously described; however, using RIPA buffer (50 mM Tris, pH 8.0, 150 mM NaCl, 1 % NP-40, 0.5 % sodium deoxycholate, 0.1 % SDS) supplemented with 10 μg/mL aprotinin, 1 μg/mL pepstatin, 1 μg/mL leupeptin, 0.4 mM Pefablock SC (Sigma). Equal amounts of soluble proteins were separated by SDS-PAGE, transferred to PVDF membranes (GE Healthcare) and blocked with 3 % BSA (GE Healthcare) PBS, 1 mM EDTA, and 0.05 % Tween 20 (blocking buffer). Primary antibodies were resolved in blocking buffer containing 0.02 % sodium azide and incubated for at least 2 h. After washing (200 mM Tris/HCl, pH 7.5, 250 mM NaCl and 0.05 % Tween 20) membranes were incubated with horseradish peroxidase-conjugated secondary antibody in 5 % milk powder in PBS for 40 min and, after washing, proteins were visualized by the ECL-system (Sigma). Signal intensities were measured using the Imaging System FluorChem 5500 and quantifications were performed normalizing signals to tubulin (AlphaEaseFC software).

### Transfection of MSC, protein expression rescue and fusion rescue experiments

MSCs were seeded on collagen-coated plates, cultured overnight, and transfected with plasmid DNA using Lipofectamine LTX with PLUS reagent (Thermo Fisher Scientific). For protein expression analyses, total cell lysates were generated 24 h later and western blots were performed as described above. In case of fusion rescue analyses, growth medium was replaced by differentiation medium 6 h post transfection. Cells were fixed with 4 % paraformaldehyde 5 days after transfection. Myoblasts and myotubes were identified by immunofluorescence microscopy using muscle-specific anti-myosin heavy chain antibody (MF-20) and Hoechst staining for detection of nuclei. Images were acquired using an AxioImager M1.2 microscope (Carl Zeiss) and processed with AxioVision Release 4.5 SP1 software. The fusion potential of was analyzed as follows: (number nuclei within fused myoblasts (≥2)/total number of nuclei) × 100. Resulting data were analyzed via two-tailed type 2 Student’s *t*-test. Transfection efficiency was approximately 35 %.

### Isolation of RNA and quantitative real time PCR

Total RNA was isolated from MSCs using TRIzol reagent (Thermo Fisher Scientific). After chloroform-extraction and isopropanol precipitation, resulting RNA yield and purity was determined with a spectrophotometer. cDNAs were obtained by reverse transcription of DNaseI treated RNA using a Revert Aid First Strand cDNA Synthesis Kit (Thermo Fisher Scientific) with oligo dT primers. Quantitative RT-PCR was performed using TaqMan Gene Expression Assays (Applied Biosystems) specific for ICln (assay ID Mm00445821_m1), MyoD (assay ID Mm00440387_m1), and GAPDH (assay ID Mm99999915_q1) for normalization to enable quantification by the comparative ddCt method. The amplification of cDNA was performed by PikoReal System (Thermo Scientific). Resulting data were analyzed via two-tailed type 2 Student’s *t*-test.

### Transient transfections and luciferase assays

MSCs were seeded on collagen-coated plates, cultured overnight and co-transfected with indicated plasmid DNA and pCMV-β-Gal using Lipofectamine LTX with PLUS reagent (Thermo Fisher Scientific). Luciferase intensities were normalized to β-galactosidase activity. Cells transfected with the equal amount of empty vector DNA served as control. Cells were harvested 48 h post-transfection in 0.25 M Tris, pH 7.5, 1 % Triton X-100 buffer, and assayed for both luciferase and β-galactosidase activities. Transfections were performed in triplicate and all experiments were repeated more than three times.

### Electrophoretic motility shift assay

The promoter region of ICln was derived from the clone P1R3 of the human genomic P1 library (Ressourcen-Zentrum/Primär Datenbank RZPD library) [26]. The sequences of oligonucleotides used for the assay (minimal essential region necessary for ICln transcription) were 5′TAGAATGTCTCATCTCTATGGTCGACGGCGTTCAGATTCCG3′ and its complementary strand 5′ CGGAATCTGAACGCCGTCGACCATAGAGATGAGACATTCTA3′ corresponding to the region spanning from –154 to –113 upstream of the first ATG codon of the ICln gene. Briefly, 1 μg of fluorescently labeled IRDye 700-labeled oligonucleotides (Metabion), representing the minimal essential region necessary for ICln transcription, was incubated with recombinant GST or GST-TIS7 proteins for 10 minutes. Where indicated, the complex was pre-incubated for 20 minutes either with a specific TIS7 antibody or with a 20-fold excess of not labeled oligonucleotides (Sigma). Samples were analyzed on a 5 % native polyacrylamide gel containing 50 mM Tris, pH 7.5, 0.38 M glycine, and 2 mM EDTA at a constant voltage of 180 V for ca. 30 minutes in TBE buffer.

### Chromatin immunoprecipitation (ChIP)

Chromatin was isolated from TIS7 WT and KO formaldehyde-treated MSCs using the EpiSeeker Chromatin Extraction Kit (ab117152, Abcam) according to the manufacturer’s protocol. ChIP analyses were carried out as described previously [44]. In brief, sonicated chromatin was centrifuged at 15.000× *g* for 10 min at 4 °C, and the supernatant (65 μg of sheared DNA per each IP) was diluted 10-fold with cold ChIP dilution buffer containing 16.7 mM Tris-HCl pH 8.1, 167 mM NaCl, 0.01 % (w/v) SDS, 1.1 % (w/v) Triton X-100, and 1.2 mM EDTA supplemented with protease inhibitors. Diluted chromatin samples were pre-cleared for 1 h with 55 μL of 50 % suspension of protein A sepharose (Protein A Sepharose CL-4B, GE Healthcare N: 17-0780-01) beads that were blocked with 0.2 μg/μL sonicated herring sperm DNA (Thermo Fisher Scientific, N: 15634-017) and 0.5 μg/μL BSA (B9000 S, NEB). Immunoprecipitations were performed using 4 μg of antibodies at 4 °C overnight. Immune complexes were collected with 75 μL of 50 % suspension of protein A Sepharose for 1 h at 4 °C followed by centrifugation at 1000 rpm and 4 °C for 5 min using a table top centrifuge. The beads carrying the immune complexes were washed once with 1 mL low salt wash buffer (20 mM Tris-HCl pH 8.1, 150 mM NaCl, 0.1 % (w/v) SDS, 1 % (w/v) Triton X-100 (Merck), 2 mM EDTA), once with high salt wash buffer (20 mM Tris-HCl pH 8.1, 500 mM NaCl, 0.1 % (w/v) SDS, 1 % (w/v) Triton X-100 (Merck), 2 mM EDTA), once with LiCl wash buffer (10 mM Tris-HCl pH 8.1, 250 mM LiCl, 1 % (w/v) sodium deoxycholate, 1 % (w/v) IGEPAL-CA630, 1 mM EDTA) for 5 min at 4 °C on a rotating wheel, and finally twice with 1 mL TE buffer (10 mM Tris-HCl pH 8.0, 1 mM EDTA). Protein-DNA complexes were eluted from antibodies by adding a freshly prepared elution buffer containing 1 % SDS and 0.1 M NaHCO_3_. The eluate was reverse cross linked by adding 5 M NaCl to a final concentration of 0.2 M and incubating at 65 °C for 4 h. Afterwards, the eluate was treated with Proteinase K at 45 °C for 1 h. The immunoprecipitated DNA was then isolated by phenol/chloroform precipitation and used as a template for real-time quantitative PCR. A primer pair (5′-ACAGCAGACGACTTCTATGATGAC-3′/5′- GTGCACCGCAGTAGGGAAG-3′) specific for region 872/1009 of the myoD gene (ENSMUSG00000009471) and (5′-TGACCTTCAAAGAGAACCACATAG-3′/5′-CCCGGAAGCAGTAGTCGTAG-3′) for the ICln promoter region were used for amplification; reactions with rabbit IgG or with 1.23 % of total chromatin (input) were used as controls. For real-time quantitative PCR a PikoReal System (Thermo Scientific) was used. Signals were normalized to input chromatin and shown as percentage input. The raw cycle threshold (Ct) values of the input were adjusted to 100 % by calculating raw Ct – log2(100/input). To calculate the percentage input of the immunoprecipitations, the equation 100 × 2[Ct (adjusted input to 100 %) – Ct (IP)] was applied.

### Surface plasmon resonance (SPR)

5′-biotinylated single strand sense and complementary non-biotinylated antisense strand oligonucleotides were purchased from Sigma. The complementary oligonucleotides were annealed in a buffer containing 30 mM Na_3_PO_4_, 200 mM NaCl, and 1 mM dithiothreitol (DTT), pH 7.2 [45].

Annealed oligonucleotides were immobilized on Sensor Chip SA (GE Healthcare) according to the manufacturer’s protocol. The sensor chip carrying the target oligonucleotide was then exposed to different concentrations of recombinant GST-TIS7 or GST alone in a Biacore™ X100 (GE Healthcare). Surface plasmon resonance signals were measured and the dissociation constant (Kd) was determined.

### Methylation assay

HEK 293 T cells (ATCC® CRL-3216™) were transiently transfected with pcDNA3.1 or pcDNA3.1 myc-PRMT5 plasmids alone or co-transfected with ICln-YFP expressing plasmid using calcium phosphate precipitation. After 24 hours, ectopic myc-PRMT5 was immunoprecipitated using anti-c-myc (9E10) antibody as previously described [46] and then incubated with 4 μg of purified core histones (Sigma) in 30 μL methylation buffer (50 mM HEPES (pH 7.9), 60 mM NaCl supplemented with 0.8 μCi S-adenosyl-L-(methyl-3H) methionine (3H-SAM) (GE Healthcare) for 1 h at 30 °C. Different amounts of the purified recombinant His-ICln were added to the methylation reactions 10 min prior the addition of radioactive SAM. Reactions were stopped by adding 2× SDS-PAGE sample buffer followed by heating at 95 °C for 5 min. Samples were subsequently analyzed by SDS-PAGE and autoradiography.

### Plasmids

Full length mouse TIS7 (mTIS7), human PRMT5 (hPRMT5, Origen, cat. no. ORIGSC116333), mouse myoD (CMV-myc-tagged myoD; Addgene plasmid 8399; principal investigator: Andrew Lassar), and human ICln (hICln) cDNA or plasmid DNA were PCR amplified and cloned in frame with the fluorescent proteins ECFP and EYFP into the pECFP-C1, pECFP-N1, pEYFP-C1, or pEYFP-N1 (Clontech) mammalian expression vectors. In addition, hICln and hPRMT5 were cloned into the bacterial expression vector pET3-His [47] for GST-pulldown assays. Murine TIS7 cDNA was excised from pcDNA3.1-HisC/TIS7 [5] and inserted into pEGFP-C1 (Clontech). YFP-tagged hICln and GST-mTIS7 were described previously [5, 48]. pGL3B luciferase reporter constructs incorporating indicated promoter regions of the human *CLNS1A* (ICln, GenBank Accession #AP000609) were described previously [26]. The promoter region of *CLNS1A* was originally subcloned into the pBlueskript SKII+ vector and a 2154 base pair region upstream of the ORF was subsequently subcloned into the dual luciferase vector pGL3-Basic (Promega) using KpnI and HindIII. Using this vector as a template, a 44 base pair region of the ICln promoter (nucleotides –156 through –113; the positions are relative to the adenine of the ORF) containing a putative TIS7 consensus binding sequence was subcloned into pGL3-Basic, again using KpnI and HindIII and named pGL3B-hIClnprom156/113. The subcloning PCR primers were: forward, 5′TACTCGGTACCTAGAATGTCTCATCTCTATGG3′ and reverse, 5′AATATAAGCTTCCGGAATCTGAACGCC3′. To engineer a vector containing two of the putative TIS7 consensus binding sequences, the same 44 base pair sequence was subcloned into pGL3B-hIClnprom156/113 with KpnI and named pGL3B-hIClnprom156/113-2x. The subcloning oligonucleotides were: forward, 5′GCATAGGTACCGCTAGCCTAGAATGTCTCATCTCTATGGTCGACGGCGTTCAGATTCCGGTACCTATGC3′ and reverse, 5′GCATAGGTACCGGAATCTGAACGCCGTCGACCATAGAGATGAGACATTCTAGGCTAGCGGTACCTATGC3′. Oligonucleotide primers were purchased from (Microsynth), and the identities of all nucleotides corresponding to the ICln promoter were verified with sequencing (Microsynth) prior to use in experiments. pRDI-shRNA-PRMT5 plasmid was generated using oligonucleotides with the sequence TAGGATCTCGGTTGGGATG based on Dharmacon Lentiviral Mouse SMARTvector Cat. No. V3SM11241-231161284.

### Retroviruses and transduction

Retroviruses and transduction were performed as previously published [49], except for the selective medium containing 2 μg/mL puromycin (Sigma-Aldrich). Cells were used for experimental analyses after at least two rounds of selection.

### Antibodies

Affinity purified α-TIS7 and α-ICln rabbit polyclonal antibodies were described previously [5, 50]. Rabbit polyclonal antibody against PRMT5 (Millipore, Cat# 07-405, RRID:AB_310589) was used for western blot in a 1:1000 dilution. Mouse monoclonal α-TIS7 (Cat# T2576, RRID:AB_477566; 1:2000), α-actinin (Cat# A5044, RRID:AB_476737; 1:2000), and α-tubulin (Cat# T5168, RRID:AB_477579; 1:2,000) antibodies were purchased from Sigma, α-GFP from Roche (Cat# 11814460001, RRID:AB_390913; 1:1000), mouse monoclonal α-ICln AA 92-201 (Cat# 611274; 1:1000) and purified mouse anti-MyoD clone 5.8A ( Cat# 554130; 1:1000) from BD Transduction Laboratories, α-6× His from Clontech (Cat# 631212; 1:5000), and α-myosin heavy chain (RRID:AB_2147781; DSHB Hybridoma Product MF 20; 1:1000) from Developmental Studies Hybridoma Bank. Affinity purified goat polyclonal α-GST was used from Pharmacia (Cat# 27-4577-01; GE Healthcare). MyoD western blot data were confirmed by additional experiments using different antibodies. Although both identified a double band (45 and 48 kDa), the specificity of these antibodies was confirmed by pre-incubation with corresponding peptides, which blocked both bands. Therefore, both bands were included for the quantification of MyoD protein levels (Fig. 2c). The Alexa Fluor488, Alexa Fluor568, and Alexa Fluor 647 conjugated secondary antibodies were purchased from Molecular Probes (Thermo Fisher Scientific).

### Statistics

Results of three experimental repeats or more (indicated as n = number of observations) are shown as indicated in the respective figure legend, either as mean ± SD or as mean ± SEM for variation between experiments. The mean values for biochemical data from the experimental groups were compared by performing two-tailed type 2 Student’s *t*-test. *P* values are as follows: *P < 0.05, ** *P* < 0.01, and *** *P* < 0.001.

## Additional files

Additional file 1: Figure S1. (**a**) TIS7 KO does not change abundance of histone H3 on the *myoD* gene. ChIP analysis using chromatin from TIS7 WT or KO MSCs. Antisera directed against histone H3 were used for immunoprecipitation. Immunoprecipitated DNA was analyzed in triplicate by qPCR using primers specific for *myoD* regulatory region. ChIP with control rabbit IgGs or 1.23 % of total chromatin (input) were used as controls. Signals were normalized to input chromatin and shown as percentage of input. (**b**) Expression of PRMT5 in control and PRMT5 knockdown cells. TIS7 KO MSCs were infected with either sh GFP- or sh PRMT5-expressing lentiviruses. Gene-specific mRNA was quantified using qRT-PCR and the comparative ddCt method. Values are presented as mean ± SD. (**c, d**) ICln does not affect PRMT5 methyltransferase activity. (**c**) Myc-tagged PRMT5 was expressed alone or co-expressed with ICln-YFP in HEK293T cells. Ectopic PRMT5 was immunoprecipitated from whole cell lysates using anti-myc 9E10 antibody and its methyltransferase activity was analyzed by in vitro methylation assay followed by autoradiography (top). Purified core histones were used as substrates. Coomassie staining shows the relative amount of histones (middle) and immunoprecipitated PRMT5 (bottom) used in the assay. (**d**) Ectopic PRMT5 was purified from HEK293T cells as described in c. The methylation of histones by PRMT5 containing immunocomplexes was analyzed in the absence or presence of recombinant purified His-ICln protein (top). The levels of histones, PRMT5, and ICln proteins were documented by Coomassie staining of SDS-PAGE (bottom). (JPG 2327 kb)
Additional file 2: Figure S2. Bioinformatics prediction software (DISIS (www.predictprotein.org), BinDN+ (http://www.mybiosoftware.com/bindn-bindn-bindn-rf-sequence-based-prediction-of-dna-and-rna-binding.html+/), DP-Bind (http://lcg.rit.albany.edu/dp-bind/) and DNABindR (http://www.imtech.res.in/raghava/dnabinder/) identified with 70–80 % probability DNA-binding domains in TIS7 amino acid sequence. (JPG 3877 kb)
Additional file 3: Raw data relating to each figure. (XLSX 236 kb)
