# Supplementary figures and images for: TIS7 induces transcriptional cascade of methylosome components required for muscle differentiation
Source: BMC Biol. 2016 Oct 25;14:95. doi: 10.1186/s12915-016-0318-6 (PMC5080701; doi:10.1186/s12915-016-0318-6)

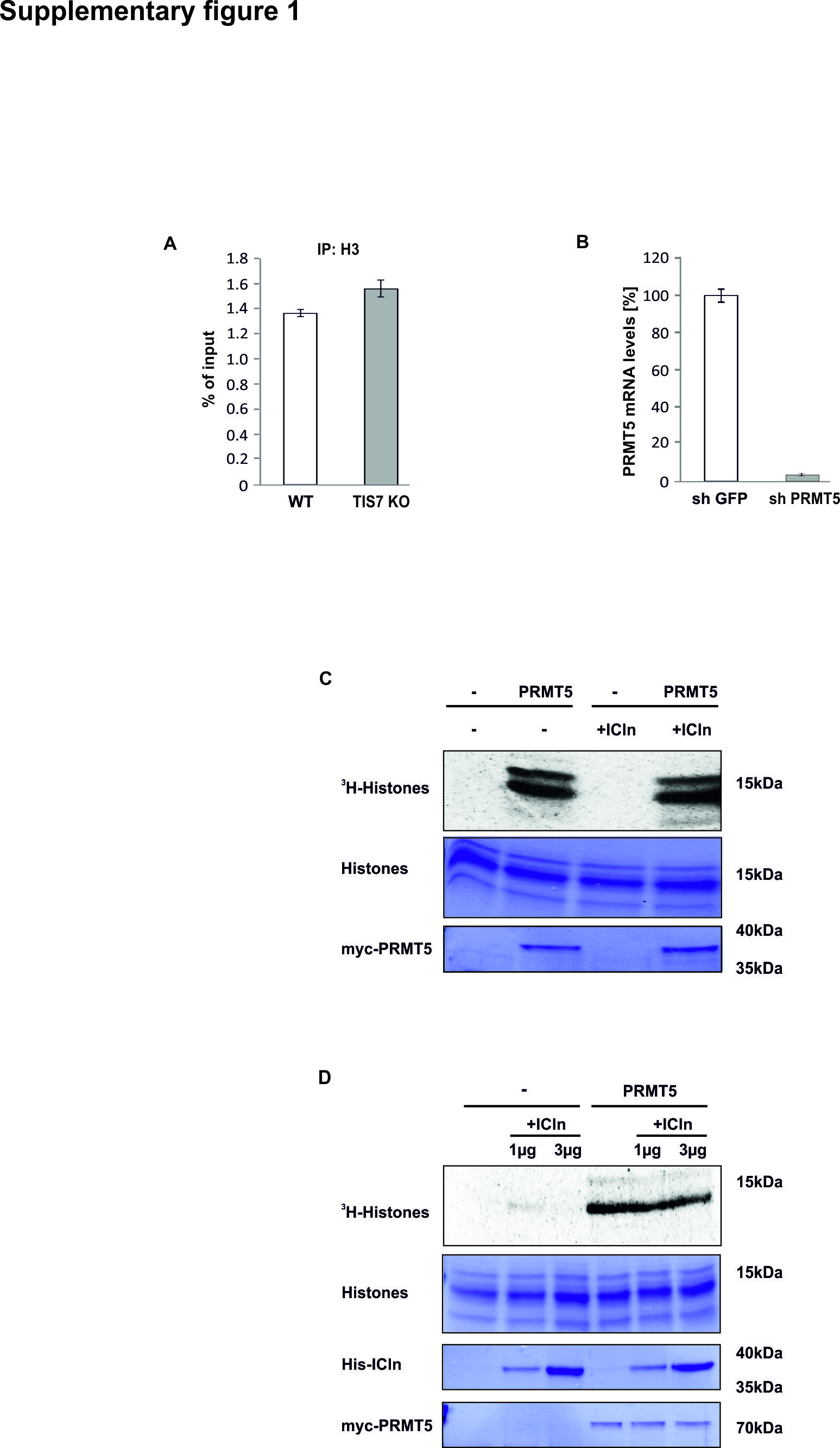

Supplement: Additional file 1: Figure S1. — (a) TIS7 KO does not change abundance of histone H3 on the myoD gene. ChIP analysis using chromatin from TIS7 WT or KO MSCs. Antisera directed against histone H3 were used for immunoprecipitation. Immunoprecipitated DNA was analyzed in triplicate by qPCR using primers specific for myoD regulatory region. ChIP with control rabbit IgGs or 1.23 % of total chromatin (input) were used as controls. Signals were normalized to input chromatin and shown as percentage of input. (b) Expression of PRMT5 in control and PRMT5 knockdown cells. TIS7 KO MSCs were infected with either sh GFP- or sh PRMT5-expressing lentiviruses. Gene-specific mRNA was quantified using qRT-PCR and the comparative ddCt method. Values are presented as mean ± SD. (c, d) ICln does not affect PRMT5 methyltransferase activity. (c) Myc-tagged PRMT5 was expressed alone or co-expressed with ICln-YFP in HEK293T cells. Ectopic PRMT5 was immunoprecipitated from whole cell lysates using anti-myc 9E10 antibody and its methyltransferase activity was analyzed by in vitro methylation assay followed by autoradiography (top). Purified core histones were used as substrates. Coomassie staining shows the relative amount of histones (middle) and immunoprecipitated PRMT5 (bottom) used in the assay. (d) Ectopic PRMT5 was purified from HEK293T cells as described in c. The methylation of histones by PRMT5 containing immunocomplexes was analyzed in the absence or presence of recombinant purified His-ICln protein (top). The levels of histones, PRMT5, and ICln proteins were documented by Coomassie staining of SDS-PAGE (bottom). (JPG 2327 kb) [file 12915_2016_318_MOESM1_ESM.jpg]

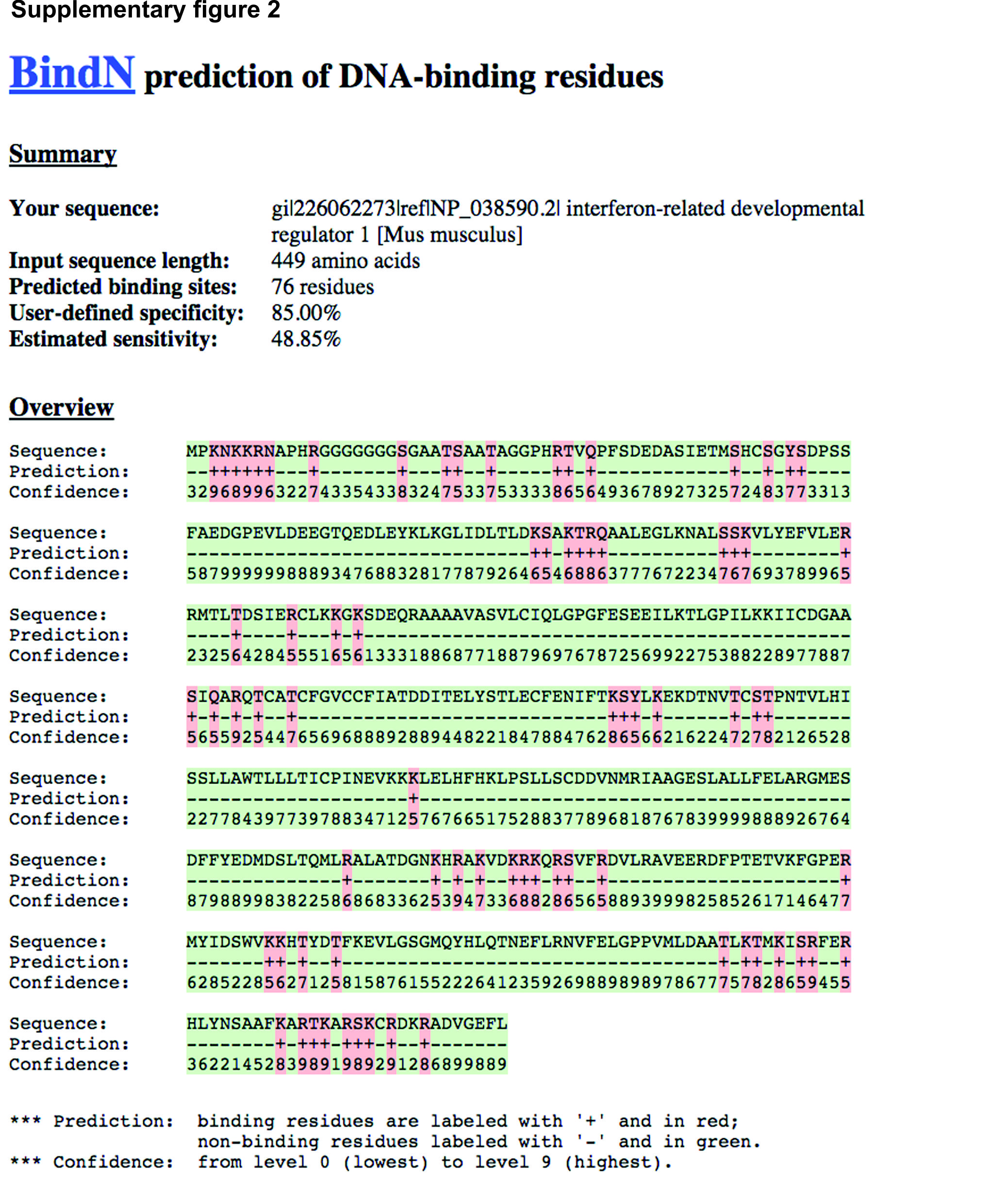

Supplement: Additional file 2: Figure S2. — Bioinformatics prediction software (DISIS (www.predictprotein.org), BinDN+ (http://www.mybiosoftware.com/bindn-bindn-bindn-rf-sequence-based-prediction-of-dna-and-rna-binding.html+/), DP-Bind (http://lcg.rit.albany.edu/dp-bind/) and DNABindR (http://www.imtech.res.in/raghava/dnabinder/) identified with 70–80 % probability DNA-binding domains in TIS7 amino acid sequence. (JPG 3877 kb) [file 12915_2016_318_MOESM2_ESM.jpg]
